# *Capnocytophaga canimorsus* Bloodstream Infection Associated with an Urticarial Exanthem

**DOI:** 10.1155/2021/9932170

**Published:** 2021-06-10

**Authors:** John C. Goetzinger, Austin L. LaGrow, Dena R. Shibib, Sharanjeet K. Thind

**Affiliations:** ^1^Department of Internal Medicine, Section of Rheumatology, Immunology, and Allergy, University of Oklahoma Health Sciences Center, 1100 N. Lindsay, Oklahoma City, OK 73104, USA; ^2^Department of Internal Medicine, University of Oklahoma Health Sciences Center, 1100 N. Lindsay, Oklahoma City, OK 73104, USA; ^3^Department of Pathology and Laboratory Medicine, Oklahoma City VA Health Care System, 921 NE 13th Street, Oklahoma City, OK 73104, USA; ^4^Department of Pathology and Laboratory Medicine, University of Oklahoma Health Sciences Center, 940 Stanton L. Young Blvd, Oklahoma City, OK 73104, USA; ^5^Section of Infectious Diseases, Medical Service, Oklahoma City VA Health Care System, 921 NE 13th Street, Oklahoma City, OK 73104, USA; ^6^Department of Internal Medicine, Section of Infectious Diseases, University of Oklahoma Health Sciences Center, 1100 N. Lindsay, Oklahoma City, OK 73104, USA

## Abstract

*Background*. Capnocytophaga canimorsus is a fastidious, slow-growing, Gram-negative rod that is a commensal bacterium in normal gingival flora of canine and feline species. Infection with the organism may cause disease ranging from flu-like symptoms to disseminated intravascular coagulation (DIC), fulminant sepsis, meningitis, and endocarditis with an overall fatality rate of 6–26%. Risk factors for infection from C. canimorsus include immunosuppression, alcoholism, and asplenia. *Case Presentation*. We describe an unusual case with a relatively indolent clinical course and an urticarial exanthem in an otherwise young immunocompetent patient with a history of type 1 diabetes. The patient presented to the Emergency Department (ED) with a <1-day history of rhinorrhea, fever, and dyspnea. He met sepsis criteria on initial presentation, but left against medical advice and returned to the ED the following day, with new arthralgias and a diffuse rash, multiple erythematous, tender macules scattered across his trunk and extremities, and tonsillar erythema. He had not taken the doses of the prescribed amoxicillin. Blood cultures two days later signaled positive for growth with the Gram stain showing a Gram-negative rod. Three 7-8 cm tender targetoid lesions with central clearing were identified on the patient's back. The patient reported two nonengorged ticks crawling on his body a week prior and sustaining a dog bite to his ear three weeks before presentation. Ultimately, the organism was identified as *C. canimorsus* through MALDI-TOF mass spectrometry and additional biochemical testing. He was given appropriate antibiotics and improved clinically thereafter. Despite the patient's bacteremia, he never progressed to fulminant sepsis and followed a mild clinical course with several unusual characteristics. *C. canimorsus* is an uncommon cause of illness in humans, but is an important pathogen to consider when evaluating a patient with a dog bite, known risk factors, and an urticarial exanthem as empiric treatment may prevent severe outcomes.

## 1. Introduction

*Capnocytophaga canimorsus* is a fastidious, fusiform, Gram-negative bacillus that comprises part of the normal oral flora of dogs and cats [1]. It was first described in 1976 after being isolated from the blood and cerebrospinal fluid of an individual who developed septicemia and meningitis following a dog bite [2]. Prior to obtaining the current name of *C*. *canimorsus* in 1989, the bacterium was classified by the CDC as DF-2 (dysgonic fermenter-2), referring to its poor, slow growth on media and fermentative metabolism [3]. Humans can acquire infection with *C*. *canimorsus* from a dog bite, with illness most commonly occurring in the settings of immunocompromise, heavy alcohol use, or prior splenectomy [1]. While *C*. *canimorsus* is a rare cause of illness in humans, it can present with severe clinical manifestations and is often fatal [4]. About half of patients infected with *C*. *canimorsus* have dermatologic findings [5]. We describe a case of *C*. *canimorsus* bacteremia that followed a relatively indolent course and was associated with an urticarial exanthem.

## 2. Case Presentation

A 31-year-old man presented to the emergency department with a 1-day history of rhinorrhea and 5-hour history of fever and dyspnea. He endorsed fatigue, postnasal drip, nausea, and mild, sharp, left-sided chest pain made worse by deep inspiration. Medical history included type 1 diabetes mellitus (hemoglobin A1c 12.8%), depression, and 17 pack-years of cigarette smoking. Upon examination in the emergency department, blood pressure was 124/74 mmHg, heart rate was 127 beats/min, respirations were 24 breaths/min, and temperature was 38.4°C. The leukocyte count was 12.4 × 10^3^/L (89.3% neutrophils), glucose 367 mg/dL, anion gap 18 mEq/L, and creatinine 0.9 mg/dL. The patient was awake, alert, and appeared in mild distress. Cardiac examination was regular and without murmurs, lungs were clear to auscultation, and abdominal exam was unremarkable. There was no oral erythema, cervical lymphadenopathy, or peripheral edema. Chest radiograph was unremarkable, and the computed tomography pulmonary angiogram was significant only for central airway thickening. The patient was administered intravenous fluids, morphine, and ondansetron. He was subsequently diagnosed with an upper respiratory tract infection and offered admission to optimize glycemic control; however, the patient declined. He was then discharged home from the emergency department with a prescription for a 7-day course of amoxicillin/clavulanate, which he did not fill.

The patient presented again to the emergency department the following day with worsening fatigue, subjective fever, arthralgias, and a diffuse rash. Vital signs were within normal limits. Examination revealed multiple erythematous, tender macules scattered across his trunk and extremities. Erythema of the right tonsillar pillar was also noted. He was offered admission for further evaluation; however, the patient again declined.

**Figure 1:**
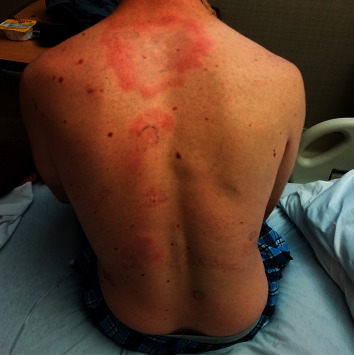
Photograph demonstrating the patient's diffuse rash with multiple targetoid, erythematous, tender macules scattered across his trunk and extremities.

Two days later, two anaerobic and two aerobic blood culture bottles obtained during his initial visit to the emergency room signaled positive. Upon Gram stain, all contained long, thin, Gram-negative rods. Each blood culture bottle was streaked for isolation on sheep blood agar, chocolate agar, MacConkey agar, and anaerobic reducible blood agar. The patient was alerted of the positive blood cultures and subsequently admitted to the hospital. On this presentation, the patient reported persistence of his rash, but otherwise felt well. Upon further questioning, the patient reported that prior to the development of the rash, he had experienced watery eyes and lesions on his oropharynx that had subsequently resolved. On admission, his vital signs were within normal limits. The leukocyte count was 12.9 × 10^3^/L (74.2% neutrophils), glucose 398 mg/dL, and C-reactive protein 59.2 mg/L. Examination of his chest revealed multiple 2-3 cm, erythematous, irregularly shaped patches that were minimally indurated and mildly tender to palpation. On his back were three irregular, 7-8 cm, targetoid lesions with central clearing that were also mildly tender to palpation (Figure 1). He was found to have tender gums along with several broken and filled teeth. The patient was started on intravenous piperacillin/tazobactam.

The following morning, the infectious diseases service was consulted for additional recommendations. Further history obtained at that time revealed the patient to have had exposure to several individuals at his school with recent international travel, although he was unaware if any had been ill. In addition, he had recently observed two nonengorged ticks crawling on his body approximately one week prior to his initial presentation. The patient also reported exposure to his pet cat and dog, both of which had fleas, and stated that he had sustained a bite to his ear by his pet dog three weeks prior to initial presentation without obvious wound infection or fever. He denied previous history of allergies, sexually transmitted infections, or new sexual partners. At this time, the rash was suspected to be associated with a tick-related illness, viral exanthem, or the Gram-negative rod growing in the patient's blood. Given the patient's poor dentition and history of a dog bite, the differential diagnosis for the Gram-negative rod included *Fusobacterium*, a HACEK organism, or *C*. *canimorsus*. The piperacillin/tazobactam was continued, and the patient was started empirically on doxycycline for possible tick-related illnesses.

On the second day of hospitalization, the patient's targetoid skin lesions had significantly faded and the lesions on his chest were beginning to resolve. Additional laboratory workup included antibodies to HIV, HIV viral load, rapid plasma reagin, fluorescent treponemal antibody absorption, viral hepatitis panel, urine gonorrhea and chlamydia nucleic acid amplification tests, *Mycoplasma* antibody, and *Bartonella* serology, all of which were negative/nonreactive. After three days, there was pinpoint growth on the chocolate agar, with Gram stain of the colony revealing faint staining, fusiform, Gram-negative rods. The isolate was catalase-positive, weakly oxidase-positive, and indole-negative. On the fifth day of hospitalization, the rash had almost completely resolved and the organism was continuing to grow on incubating plates, but did not grow on selective media. The Vitek2 NH card (Biomerieux, Durham, NC) identified the isolate as either *Neisseria elongata* or *N*. *gonorrhoeae* with low discrimination. The isolate was retested with an 85% probability of *N*. *elongata*. The Vitek2 NH card contained biochemicals for identifying *Capnocytophaga* species, but that was not listed as a possible identification for this isolate. The patient was discharged home on hospital day 4 with intravenous ceftriaxone, oral metronidazole, and a 14-day course of oral doxycycline to cover for potential tick-borne related infection. However, two days after discharge, the patient called the hospital reporting similar symptoms to what he had experienced on initial presentation. The ceftriaxone and metronidazole were discontinued at this time and he was switched back to piperacillin/tazobactam. Due to the uncertainty of the bacterial identification, the isolate was referred to another laboratory which used a combination of biochemical testing and matrix-assisted laser desorption ionization time-of-flight mass spectrometry (MALDI-TOF MS; Bruker, Billerica, MA) to identify the Gram-negative rod as *Capnocytophaga canimorsus* with a log score value of 2.59. Antimicrobial susceptibility testing performed by the E-test revealed MIC values of 2 mcg/mL for ceftriaxone and 0.03 mcg/mL for piperacillin. No current breakpoint interpretations are available; however, based on the literature, similar MIC values for these antibiotics were associated with clinical improvement [6]. The patient improved clinically thereafter and remained in good health for two years following the infection.

## 3. Discussion

Of the nearly 500 documented cases of *C*. *canimorsus* infection as of 2014, the fatality rate was approximately 6.2% to 26% [1, 7, 8]. A variety of clinical presentations have been described, with the most common being sepsis. Many patients develop shock, disseminated intravascular coagulation (DIC), purpura or petechiae, peripheral gangrene, and gastrointestinal symptoms [1]. Other reported clinical presentations include meningitis, endocarditis, cellulitis, and eye infections [1]. Infections with the organism are typically treated with beta-lactam/beta-lactamase inhibitor, 3^rd^/4^th^ generation cephalosporins, or carbapenems [6, 7]. Our case illustrates a less common presentation of *Capnocytophaga* infection presenting with mild constitutional symptoms and an urticarial exanthem in a patient with history of a dog bite that occurred several weeks prior to the patient first seeking medical attention, diabetes, but no specific immunocompromising factors. Important risk factors for *C*. *canimorsus* infection in humans are dog bites, contact with dogs, immunocompromised state, heavy alcohol use, and prior splenectomy or hyposplenism [1]. Cat bites and exposure to cats have also been implicated as risk factors but are much less common [1]. This pathogen more commonly affects male patients between 50 and 60 years of age, with an incubation period between animal exposure and symptom onset of 7 days, on average [8]. Taking this information into account, our patient's presentation was unique in several ways. In contrast to most patients with documented *C*. *canimorsus* infection, our patient's clinical features were overall much less severe. Despite his bacteremia, the patient did not meet sepsis criteria or develop other complications with which *C*. *canimorsus* is known to be associated, nor did he possess any of the traditional risk factors associated with this infection other than exposure to his pet dog, subsequent bite, and an immunocompromised state due to uncontrolled type 1 diabetes mellitus. Additionally, his relatively young age at presentation and the three-week incubation period from the time of dog bite to symptom onset have rarely been reported.

To the best of our knowledge, there have only been three previous cases of urticarial exanthem associated with C. canimorsus infection described in the literature, two of which were associated with a dog bite and the other had exposure to pet dogs, but no known dog bite. All three patients had positive blood cultures for *C. canimorsus*. One of the patients had type 2 diabetes mellitus as well as alcoholism. Interestingly, one of the patients recovered without any antibiotics [5, 9, 10].

## 4. Conclusions

Although *C*. *canimorsus* is a relatively rare cause of illness in humans, it is an important pathogen to consider when evaluating a patient with a dog bite and known risk factors, as empiric antimicrobial therapy could potentially prevent adverse outcomes. The association of urticarial exanthem with *C*. *canimorsus* infection may also prove to be equally important for clinicians to recognize.

## Data Availability

The data used to support the findings of this study are presented in this case report; additional information from the case is available through the corresponding author upon request.
